# Developing a Health Support System to Promote Care for the Elderly

**DOI:** 10.3390/s25020455

**Published:** 2025-01-14

**Authors:** Marcell Szántó, Lehel Dénes-Fazakas, Erick Noboa, Levente Kovács, Döníz Borsos, György Eigner, Éva-H. Dulf

**Affiliations:** 1 Biomatics and Applied Artificial Intelligence Institute, John von Neumann Faculty of Informatics, Obuda University, 1034 Budapest, Hungary; mszanto@stud.uni-obuda.hu (M.S.); denes-fazakas.lehel@nik.uni-obuda.hu (L.D.-F.); kovacs@uni-obuda.hu (L.K.); 2 Physiological Controls Research Center, University Research and Innovation Center, Obuda University, 1034 Budapest, Hungary; 3 Applied Informatics and Applied Mathematics Doctoral School, Obuda University, 1034 Budapest, Hungary; noboa.erick@uni-obuda.hu; 4 Institute of Instrumentation and Automation, Kandó Kálmán Faculty of Electrical Engineering, Obuda University, 1034 Budapest, Hungary; borsos.doniz@uni-obuda.hu; 5 MedTech Innovation and Education Center, University Research and Innovation Center, Obuda University, 1034 Budapest, Hungary; 6 Department of Automation, Faculty of Automation and Computer Science, Technical University of Cluj-Napoca, 400114 Cluj-Napoca, Romania

**Keywords:** dementia, Alzheimer’s disease, elderly, healthcare, safety, LoRaWAN, biometrics identification, PPG, fall detection

## Abstract

In light of the demographic shift towards an aging population, there is an increasing prevalence of dementia among the elderly. The negative impact on mental health is preventing individuals from taking proper care of themselves. For individuals requiring hospital care, those receiving home care, or as a precaution for a specific individual, it is advantageous to utilize monitoring equipment to track their biological parameters on an ongoing basis. This equipment can minimize the risk of serious accidents or severe health hazards. The objective of the present research project is to design an armband with an accurate location tracking system. This is of particular importance for individuals with dementia and Alzheimer’s disease, who frequently leave their homes and are unable to find their way back. The proposed armband also includes a fingerprint identification system that allows only authorized personnel to use it. Furthermore, in hospitals and healthcare facilities the biometric identification system can be used to trace periodic medical or nursing visits. This process improves the reliability and transparency of healthcare. The test results indicate that the armband functions in accordance with the desired design specifications, with performance evaluation of the main features including fall detection, where a hit rate of 100% was obtained, a fingerprint recognition test demonstrating accuracy from 88% to 100% on high-quality samples, and a GPS tracking test determining position with a difference of between 1.8 and 2.1 m. The proposed solution may be of benefit to healthcare professionals, supported housing providers, elderly people as target users, or their family members.

## 1. Introduction

Today, due to the presence of aging societies, there are an increasing number of people with old-age dementia. This is mainly caused by various diseases, the most common of which are Alzheimer’s disease and dementia [1,2,3,4]. According to Alzheimer’s Disease International, there were over 55 million people with Alzheimer’s disease worldwide in 2020 and it is expected that this number will almost double every 20 years [5]. In Hungary, the dementia in the population is increasing. In fact, more than 200,000 inhabitants were diagnosed with dementia between 2011 and 2016—the numbers have been growing in recent years as well [6]. According to another survey, the number of people with dementia in Europe is estimated at 9.1 million. In countries where society is aging, such as Hungary, the number of people with dementia is higher. In Hungary, the number of people affected is 145,000 [7].

**Figure 1:**
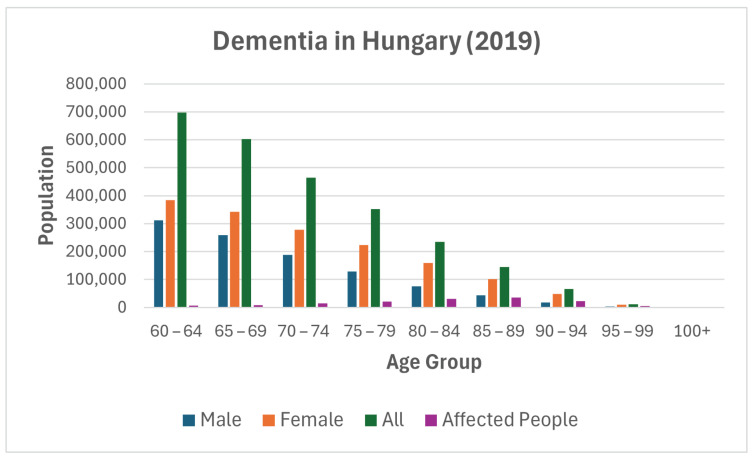
Estimated dementia in Hungary 2019 [7].

Figure 1 shows the estimated number of people with dementia living in Hungary during the year 2019 [7]. The total population per age group is represented in green and the subgroup of affected people with dementia is shown in purple.

In addition to the diseases mentioned above, there are many other diseases that can negatively affect people’s mental health resulting in people being unable to take proper care of themselves [8]. Whether in the context of hospital care, in the context of a home care system, or only as a precaution for a particular person, it is beneficial to use some means to help monitor their biological parameters on an ongoing basis. Use of such equipment can reduce the likelihood of serious accidents or severe health hazards. A 2007 WHO (World Health Organization) survey revealed that one in three individuals aged 65 and above experience a fall annually, while one in two individuals aged 70 and above fall once a year. This is the same age group that has a higher incidence of dementia or Alzheimer’s disease [9]. These accidents can often result in upper limb injuries, femoral neck fractures, cranial fractures, or lower hip fractures, with 20% of those in need of hospital care never leaving the institution again. Asociated with this, a fall is one of the most common causes of accidental death [8]. Furthermore, in 2006, the cost of outpatient care for hospital admissions resulting from falls was approximately EUR 2’427.247.00, while the cost of care for hospital patients was between EUR 85 and 90 million. This poses a significant financial burden on the health care system [9]. In addition to these effects, falls have another aspect that is at least as damaging as the physical injury itself—the fear of falling again. Those who have once been injured by a fall can be afraid to travel alone, leading to narrowing of their living space and deterioration in their social relationships, and thus deterioration in their quality of life. The quality of life of those affected could be improved with a device that calls for help immediately if they get into trouble. For example, in the event of a fall, to reduce damage to health, it is crucial that help arrives quickly.

The present research proposes the design of an armband with the capability of determining the location of a person with an accuracy of 2 m, which is of particular importance for individuals with dementia and Alzheimer’s disease, who frequently leave their homes and are unable to find their way back. A body-worn device capable of location tracking is crucial to avoid the aforementioned cases and reduce the risk of injuries in patients, especially for those who suffer from an elder illness [10]. The proposed HealthCare and Safety armband 2020 includes a fingerprint identification system that allows the armband to be used only by an authorized person. Furthermore, in hospitals and healthcare facilities where multiple people are treated at the same time, not all patients visit. The biometric identification system on the device can be used to trace such periodic medical or nursing visits. In a home nursing system, the nurse or doctor need only to touch the sensor on the armband during the patient’s visit and registration takes place. In this way, the reliability and transparency of healthcare can also be improved.

There are tools already available on the market that provide some solutions to the problems mentioned. Examples of such devices are the Xiaomi MiBand 5, the Philips Health Watch, and the Garmin Vivoactive 4S, which can be used to track an individual’s heart rate change for up to several days [11,12]. The Asus VivoWatch SP HC-A05 watch provides ECG (Electrocardiogram) and PPG (Photoplethysmography) measurement during wear [13,14]. The Fall Detector watch, developed by CareLink Monitoring in Ireland, can detect a fall and call for help automatically [15]. Current civilian-purpose devices, e.g., smartphones and fitness trackers, rely on global navigation satellite systems for their portability (GNSS) [16]. These devices’ performance depends on a variety of factors, i.e., the number of satellites available, their geometry and the weather conditions, with an average tracking accuracy of 4.9 m (16 ft) provided [17].

The development presented in this paper aims to address the scenarios mentioned previously by gathering and even enhancing some of the capabilities that currently available devices can offer individually, i.e., fall detection, alerts/notifications, position tracking or biometric security (see Section 4 for a more detailed comparison). The paper is structured in five sections. After this introductory part, Section 2 describes the materials used and the methods implemented, with the results presented in Section 3. Section 4 is dedicated to a discussion and comparison with related findings. The work ends with a concluding section.

## 2. Materials and Methods

### 2.1. Architecture Design the Armband Contains Five Main Parts [18]

These are LoRa S76G, GY-521, Mikroe Fingerprint Click, Mikroe Heart Rate 8 Click and STM32 L412KB MCU. The operating principle and technological background of the devices are detailed in the following discussion.

### 2.2. Embedded Equipment and Tools

**Microcontroller:** The MCU is an ultra-low-power device with many communication interfaces. It is based on the Arm Cortex-M4 core and offers a good balance of performance, power efficiency and features. The MCU is equipped with a variety of communication interfaces, e.g., USB 2.0, I2C, ISART, SPI and others, to connect with various sensors, actuators and other devices [19].

**Figure 2:**
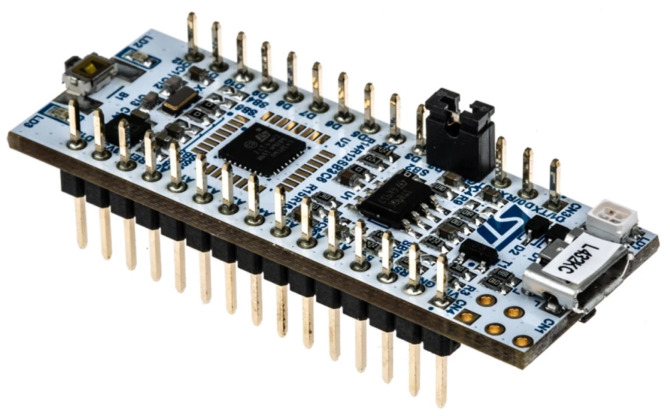
STM32L412KB microcontroller [20].

In terms of communication, the I2C (I2C is a serial communication protocol) is most important for the device because Heart Rate 8 Click and GY-521 use this as well. The LoRa communicates with the MCU (Microcontroller Unit) via UART (Universal Asynchronous Receiver-Transmitter), and the Fingerprint Click uses SPI (Serial Peripheral Interface). No large computing capacity is required during the planned operation; thus, the MCU represents a good choice based on the ARM CORTEX M4 it contains. Furthermore, the STM provides a development environment for hardware such as STM32CubeMX in GPIO (General-Purpose Input/Output) settings or STM-STUDIO-STM32 in debugging. The present research employs these software tools for development purposes, with the code required for the operation being written in the embedded C language. The MCU module used is shown in Figure 2.

**LoRa module: [21]** LoRaWAN is a relatively often used technology in IoT [22], because it allows a large amount of data to be transferred with low energy consumption. In addition, the device can communicate over long distances thanks to frequency sweep modulation [23]. For these reasons, the device uses LoRaWAN communication. There is an available network service for LoRa, e.g., loriot.io. This allows us to use the technology easily and transparently. A LoRa device can work with three types of settings:

- Class A:
  - A device can send an uplink message at any time. Once the uplink transmission is completed the device opens two short receive (downlink) windows;
  - Device can only be addressed during a specific time;
  - Most energy efficient.

- Class B:
  - Usually one-way communication;
  - Device can be addressed on scheduled time;
  - Usually used in non-time-critical processes.

- Class C:
  - Two-way communication;
  - Device can be addressed at anytime;
  - Usually used in process controls.

**Figure 3:**
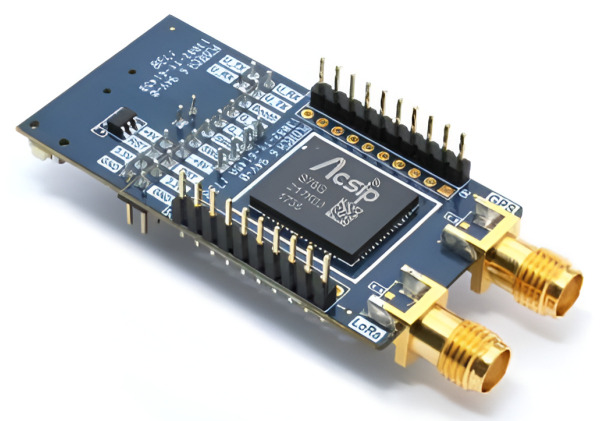
LoRaS76G module [24].

This research used the LoRa device in class C because the addressability at anytime is important in a medical device. The LoRa module used is shown in Figure 3. In device settings, it is possible to set a sleep mode. Thus the energy consumption is more favorable. In Europe, a LoRa device can be used without a radio license in the 433 MHz or the 868 MHz frequency bands. The 868 MHz (ISM—frequency band for Industrial, Scientific and Medical applications)) frequency band was chosen because at lower frequency, the output power is limited to −22.4 dBm. The same feature on 868 MHz is −1.25 dBm. The calculation of the transmission power is shown in Equation (1), where P indicates power on the sender side.

(1) $$\begin{array}{c} P\left(\mathrm{mW}\right)=1\;\mathrm{mW}\cdot 10^{\frac{-1.25\;\mathrm{dBm}}{10}}\\ P(\mathrm{mW})=0.749 \end{array}\;$$

The selected LoRa S76G contains a SONY CXD5603 GPS chip, which enables GPS-based positioning. An antenna other than the communication antenna is required to receive these signals; therefore, at the top of the module are two SMA (Surface Mount Assembly) antenna connectors. GPS (Global Positioning System) coordinates are also transmitted over the LoRa network, and the format of the message in the device settings can be specified. The IPSO (Internet Protocol Security Option) format was used because it can be easily converted to real coordinates. The first 2 bit indicates the data channel, while the following 2 bit indicates the type of the message. This is followed by the co-ordinates in terms of a 5-bit latitude, 5-bit longitude and 5-bit altitude represented in a hexadecimal number system. After separation, it is possible to convert the numbers from the hexadecimal number system to a decimal number system; the obtained values are divided by 1000, and then the coordinates are obtained. A Kerlink iFemToCell gateway was used to receive signals sent from the LoRa module. The LoRa S76G is positioned on the edge of the electronics in the armband to minimize high-frequency noise from other devices. Table 1 shows how a message recieved from the LoRa GPS module is decoded [21].

**Heart Rate Click: [25]** The measurement of pulse can happen in several ways. The most popular and reliable method is photoplethysmography. This method is based on the light-absorbing ability of blood. It depends significantly on the oxygenation of the blood. If we illuminate blood with infrared light, we can deduce the oxygenation of the blood from the absorbed and reflected light. The oxygen content of the blood in the illuminated capillaries varies in proportion to the heart rate. Thus, we can measure the pulse.

**Figure 4:**
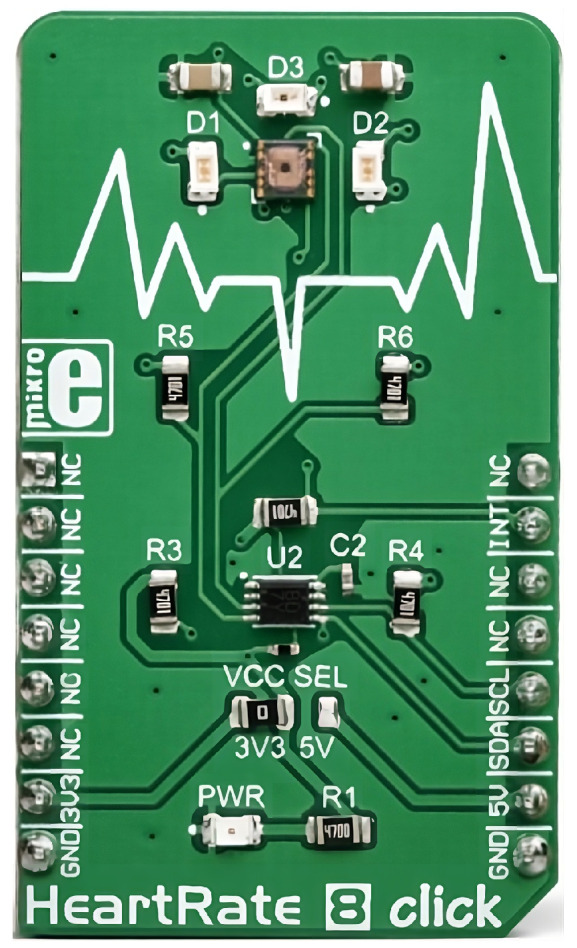
Mikroe Heart Rate 8 Click biosensor [26].

The Heart Rate 8 Click is an optical biosensor, which is excellent for pulse measurement. The device is shown in Figure 4. The module contains three LED (two green and one infrared) and one light sensor (BH1792GLC), which is sensitive to green and infrared light between 700 nm and 900 nm. The device can operate in three different measurement modes:

- synchronized measurement mode;
- not synchronized measurement mode;
- simple measurement mode.

The measured values are placed in a FIFO (First In First Out) in synchronized mode, but the appropriate registers require to be read immediately in the other two measurement modes. The Heart Rate Click 8 is placed on the inside of the armband and measures on the lower, inner part of the upper arm.

**Figure 5:**
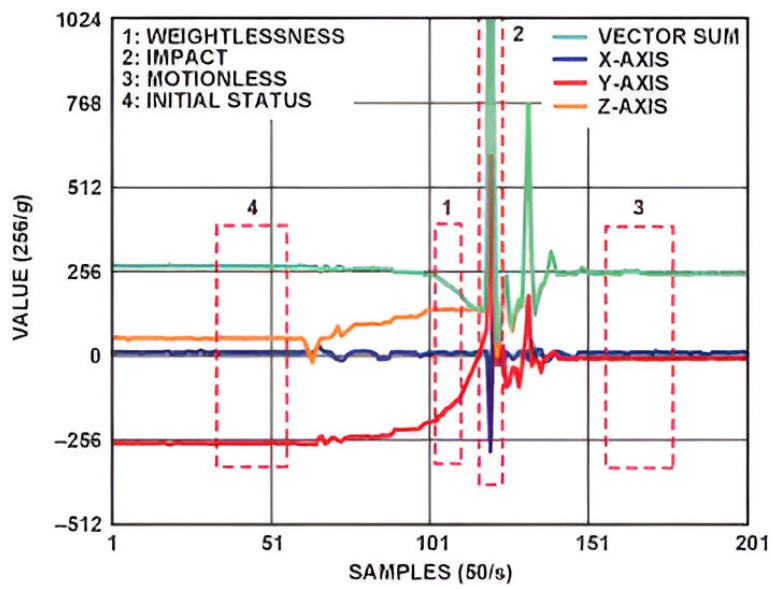
Acceleration data on 3 axes and phases of fall [28].

**Acceleration and rotation sensor: [27]** The process of falling can be divided into three stages. The three main states, supplemented by the initial status, are shown in Figure 5. The first stage is Free-fall, where the vector sum of acceleration converges to 0 G, where G is the gravitational pull of the Earth. The convergence time to 0 G depends on from how high the fall started. In the case of a normal fall, we are not talking about real free-fall but, nevertheless, the vector sum of acceleration should be less than 1 G (see section “1” in Figure 5). The second stage of falling is the Activity stage, in which a sudden change of speed occurs due to a collision with the ground or an object (see section “2” in Figure 5). Recognizing this shock, we are able to detect the second phase of the fall. The third stage of the fall is the Inactivity stage. After landing or colliding, people are generally unable to stand up immediately. This period is characterized by a short period of immobility. This is indicated by a short and flat section on the acceleration curve (see section “3” in Figure 5). In addition to these, the direction of the individual’s body also changes from the beginning, which provides useful data by means of the gyroscope. The combination of these phases constitutes the fall detection algorithm. The time between different events must be within a specified limit. For example, the time between the Free-fall stage and the Activity stage would not be very long.

To measure the acceleration and rotation, a GY-521 module was used, which contains an MPU6050 3-axis accelerometer and a 3-axis gyroscope. The device contains a 1024-byte FIFO buffer, which collects data when the device is in stand-by mode. The collected data are processed when the device is turned on.

**Fingerprint Reader: [29]** There are basically 3 ways to identify a person:

- knowledge base;
- object base;
- biometrics base.

**Figure 6:**
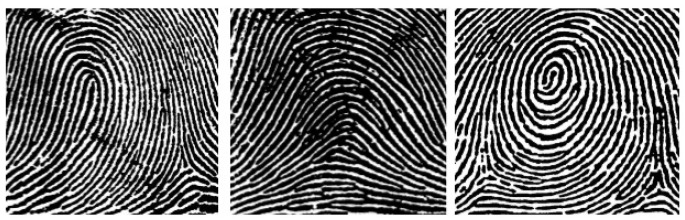
Vault, loop, swirl ruffled fibers pattern [33].

Of the three types of identification, biometric identification is the most reliable. This method uses a physical or biological feature of the human body that can be interpreted by machines, e.g., the uniqueness of the fingerprint, the ear or the retina [30]. In contrast to the other two methods, it is not the tool or information that is the basis for identification, but the person to be identified. This guarantees that the property on which the identification is based cannot be stolen. The purpose of biometric systems is to determine whether the person to be identified is the same as a person already in the database. In the case of authentication, this means comparison with a specific sample, while in the case of identification, the system must quickly search the database for the matching person or indicate that the person sought is not in the database. The features extraction process differs for different biometric sources. In the case of fingerprint identification, the goal is to find different patterns on the bitmap obtained from a given sample, extract the characteristics of the mentioned patterns, and compare them against the ones in the database [31]. The comparison result retrieves the calculation of an index of similarity between two samples. This similarity index is a distance function interpreted based on the characteristic, which is defined in terms of the distance in space of the characteristic [32]. The uniqueness of the fingerprint is reflected in the pattern of folds and grooves on the surface of the skin. These folds, also known as ruffled fibers, form different patterns that can be divided into larger units, such as vaults, vortices and loops [32]. These are shown in Figure 6.

**Figure 7:**
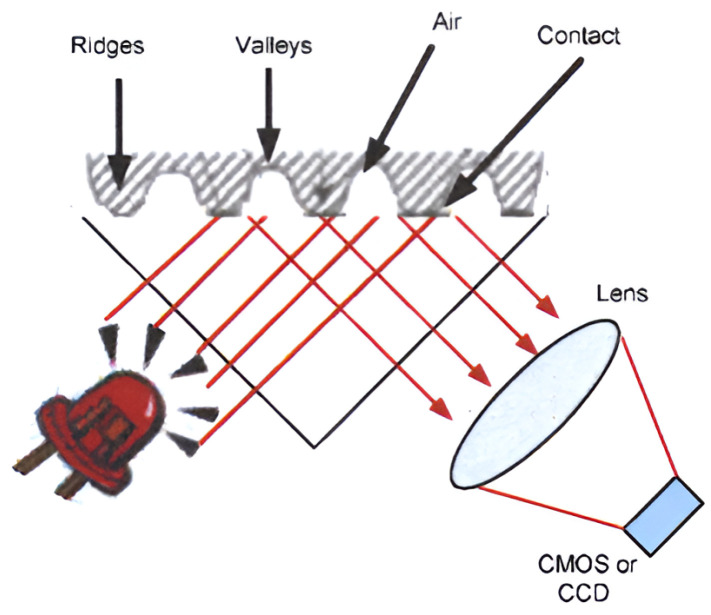
Optical fingerprint reader operation [38].

Within the larger patterns mentioned above, the present research distinguishes smaller characteristic points of the given finger relating to the ends, branches, or intersections of the ruffled fibers. Examples of such characteristic points include hooks, ends, points, islands, bridges, branches and intersections [34]. The branchings, ends, or confluences of ruffled fibers are also called minutiae points. There are several technical solutions for scanning fingerprints, in which the similarity is that each examines the uniqueness of the ruffled fiber patterns found on the fingers [35]. There are multiple technologies applied in optical devices, i.e., chip sensors, diffraction gratings and total internal reflection (TIR) sensors. With this technology, the fingerprint to be processed is imaged on the surface of an image resolution device (complementary metal oxide semiconductor (CMOS): a semiconductor device that serves as an “electronic eye”), or with a CCD element (charge-coupled device: a light-sensitive integrated circuit that captures images by converting photons to electrons), using an optical system (prism, Fresnel lens). There are also non-optical technologies that are applied in identification sensors, including those based on thermal analysis, or radio frequency, ultrasound, capacitive, or pressure sensor principles [31,36]. Devices operating on these principles create a fingerprint image from different pressure measurements, capacity values or reflected waves [37]. Among the devices operating on the optical principle, the GTS-511E2 based on chip sensor technology was chosen. The operational principle of the optical sensor is shown in Figure 7, in which a finger is placed directly on the surface of the sensor, and the information to be processed is guided to the image resolution device with optical fibers. This solution is distortion-free, provides good quality results, and takes up little space.

This type of sensor includes a CMOS (complementary metal oxide semiconductor) or CCD (charge-coupled device) detector array and a fiber optic front panel. The fiberglass front panel consists of short optical fibers arranged perpendicular to the plane of the CMOS detector array. In use, the individual places the finger on the front panel and the residual light is reflected at the sensor contact points, i.e., the backbone of the individual’s fingerprint is conducted by optical fibers directly to the CMOS detector array or CCD, where the individual’s fingerprint image is formed. The sensor uses artificial light to capture a digital image of the fingerprint pattern. To distinguish between 2-dimensional images and real fingerprints, the CMOS image sensor has a special lens and cover. As the surrounding of the sensor becomes dark when a person presses their finger against the lens, an LED illuminates the objective from one side, as described in Figure 7.

**Biometrics Identification System:** The image obtained from the sensor is very dark and contains unnecessary information; therefore, it is not yet suitable for highlighting features [18]. Consequently, the incoming image must be processed first, based on the following procedures [39]:

- Grayscale: If colors are not important, they are just noise. In addition, processing a gray-scale image takes a quarter of the time to process an RGB image, and the dominant shades can be highlighted much better in a gray image.
- Ridge detection: The ruffles are highlighted in this step, identifying the raised lines or patterns on the surface to provide the basis for the minutiae.
- Enhancement: The essence of contrast enhancement is that by examining each pixel, it shifts to a more intense hue of its own color, depending on its value. In this case, the incoming image is grayed out, so by shifting the colors toward a more intense hue, it also does most of the noise filtering. This is necessary so that noises can be recognized later as minutiae.
- Binarization: Its operation usually involves comparing the value of the current pixel with some threshold number. If the value of the examined pixel is below the threshold number, it will be white after binarization; if it is above the threshold value, it will be black. The present work improves the efficiency of the binarization operation by using a histogram.

(2) $$B\left(i,j\right)=\left\{\begin{array}{cc} 0\;if & I(i,j)<T\\ 1\;if & I(i,j)\geq T \end{array}\right.\;$$

**Figure 8:**
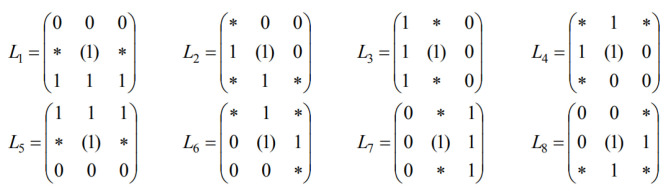
Golay alphabet masks [40].

- Fingerprint thinning: Thinning objects is possible by selecting and removing pixels at the edge with the hit-or-miss transformation, i.e., subtracting them from the original object. Sequential thinning can be accomplished by sequential application of Golay alphabet masks. The Golay alphabet masks are show in Figure 8.

- Minutiae search: The method of searching for minutiae is to look at all 8 neighbors of a given pixel along a line thinned to 1 pixel wide. Taking the sum of these pixels, if this sum is equal to zero, there are no minutiae. In contrast, if only one of the adjacent points exists, it is an end, and if two exist, it is a branch.

**Figure 9:**
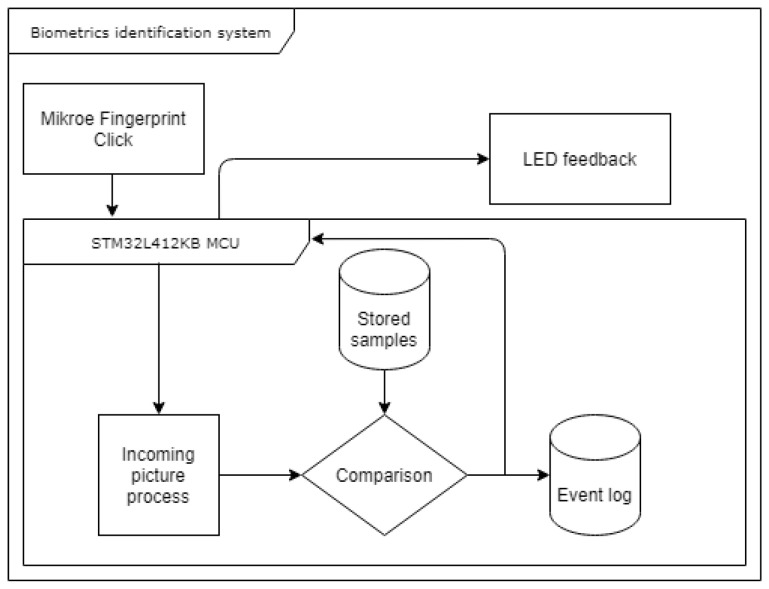
Biometrics identification system plan [18].

**Figure 10:**
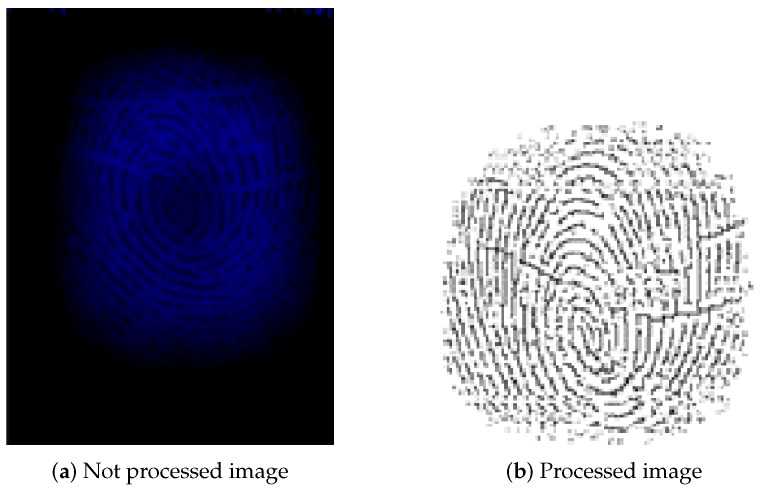
The fingerprint before and after processing.

The created system stores the location, direction, and type of minutiae found. It will make a comparison with these later. A block diagram of the fingerprint identification system in the armband is shown in the Figure 9, while Figure 10 shows the difference between the raw data obtained by the sensor (a), and the resultant image after being processed (b).

**Figure 11:**
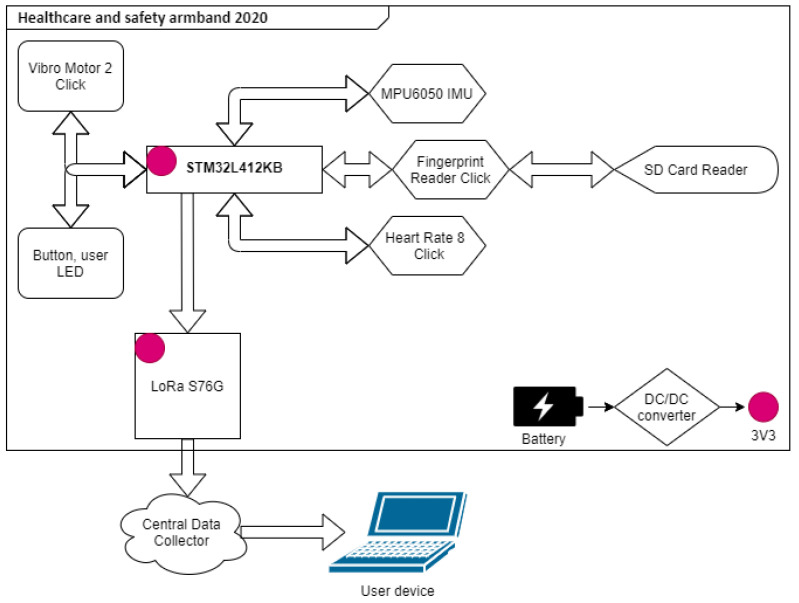
Healthcare and Safety armband 2020 system plan [18].

**The whole system and power consumption:** The logical system plan containing the structure of the entire system is shown in the Figure 11. Each peripheral is connected to the STM32 microcontroller acting as a central hub.

Battery selection was based on preliminary independent consumption measurements and calculations, as described in Table 2, where an average current drain of 41.45 mA was estimated taking into consideration the consumption values of each peripheral in different scenarios based on their datasheets. In addition, as a safety factor, the consumption was set to 50 mA instead of the calculated rate. The immersion tests were performed by simply turning on the device and using it continuously.

Currently, the system is powered by a 3500 mAh battery cell (considering the required capacity but also the space available inside the armband), which provides an average of 64.41 h of continued operation for the armband. The power consumption of each component is indicated in Table 2, and Section 3 contains a more detailed description of the tests applied to the entire device.

A deeper analysis of the current consumption of the proposed armband is planned for further developments of the device.

The total power consumption of the device is approximately 40 mA according to calculations. As this is an estimation based on independent device consumption measurements, with a safety margin, the maximum power consumption of the device was set to 50 mA for further calculations. Taking in consideration that the ideal operational time for the device is 72 h, a battery with a minimum capacity of 50 mA × 72 h = 3600 mAh is required [41] to achieve the planned goal for future development.

### 2.3. Node-RED Software System

Node.js is a software system designed to build web servers. The programs can be written in JavaScript and the system has available a display interface where the programmer can show data in different representation formats. This dashboard is used to display the coordinates of the health status and location of the person wearing the device. In Node-Red, a first instance was created in a section that connects to a web socket on a LorIoT network [42]. When the data sent by an endpoint arrives in this, they are converted into a JSON (JavaScript Object Notation is a lightweight data-interchange format.) object, which is processed to extract the main information from it. If this is the coordinate of the data, it is subdivided, converted to a decimal number system, counted back in degrees and then added to the map parameters used to indicate the location. The user interface image is shown in in the following section.

## 3. Results and Discussion

Several tests were carried out on different parts of the armband, i.e., the range and reliability of the LoRa module, the reliability of fall detection and the fingerprint identification system were tested, as well as the functionality of the armband. Prior to the aforementioned tests, verbal consent was obtained from the users involved. The subjects were students between the ages of 23 and 26, comprising both male and female participants. All tests were conducted on the university campus, in the laboratories and canteen. For the fall detection test, the subjects were observed to perform a total of 40 events, with each event occurring 10 times. These events included sitting on chairs, throwing themselves down on beds, sitting on a mattress on the floor, and acting as if they were falling. As another test, a total of 368 min of sound data were recorded for the purpose of analyzing the sounds made during the consumption of food in the canteen and other locations on campus during lunch and dinner. In total, 40 + 31 measurements were used for the meal detection test, which was conducted using the IMU section only.

**Figure 12:**
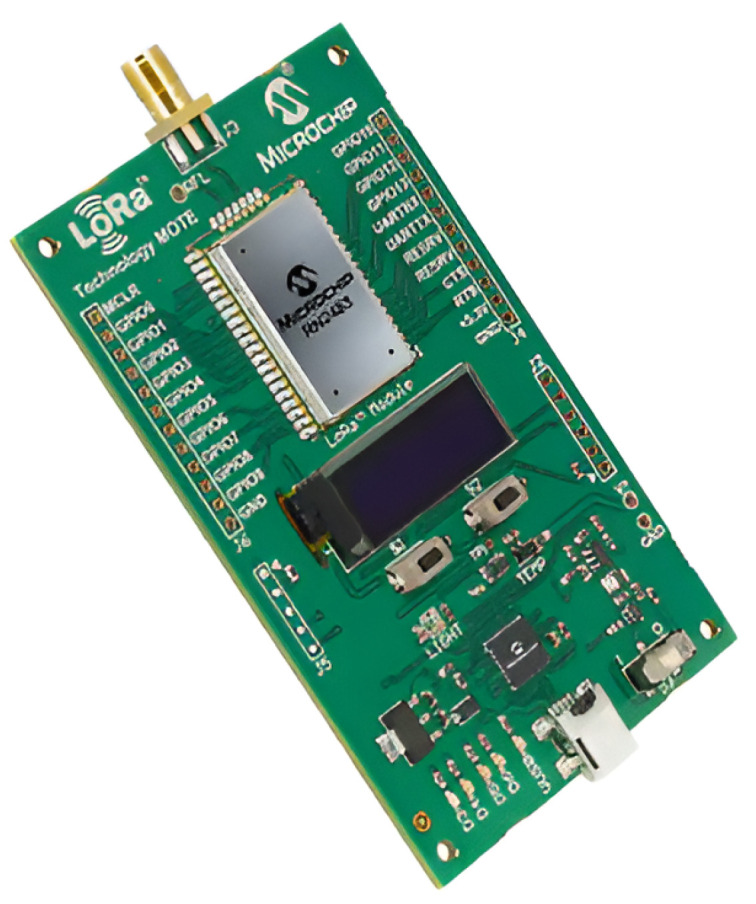
LoRa RN2483 Development Kit [43].

**Figure 13:**
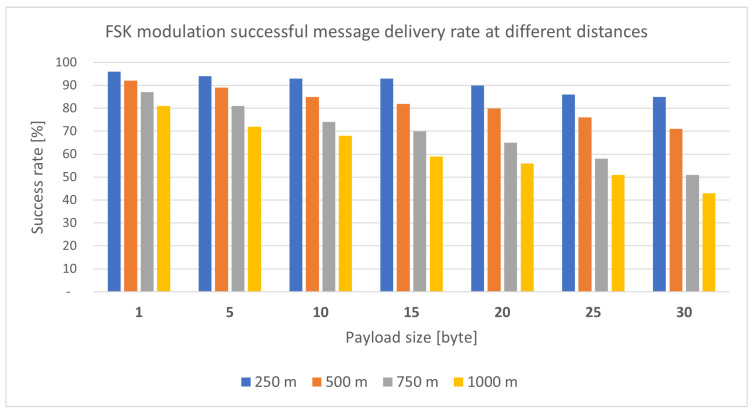
Successfully sent packets rate with FSK modulation [18].

**Figure 14:**
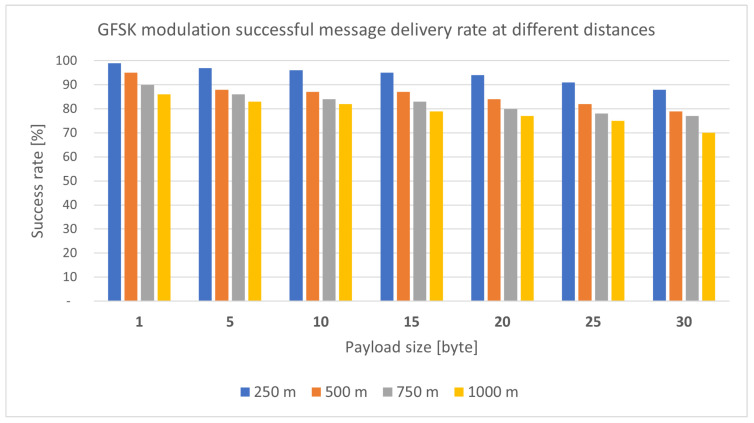
Successfully sent packets rate with GFSK modulation [18].

**LoRa module test:** For a medical armband, a critical factor is the range within which the device has reliable coverage. Therefore, it was a priority to investigate the reliability of LoRa communication, in this case based on a module RN2483 shown in Figure 12, as a function of distance and payload size in the case of FSK (Frequency-shift keying modulation) and GFSK (Gaussian FSK modulation) modulation, as can be noticed in Figure 13 and Figure 14, respectively. The mentioned tests were performed in February 2024, in clear weather on Margaret Island (Budapest, Hungary). Measurements with the same parameters are likely to produce different results under other weather and terrain conditions. This still needs to be investigated in the future.

The bar chart illustrated in Figure 13 and Figure 14 clearly shows that for both modulations, the number of successfully sent packets continuously decreased as the distance increased. The same is true for the increase in useful data. In the case of GFSK modulation (Gaussian frequency-shift Keying) Figure 14, minimally better results were obtained.

The parameters applied in the LoRa transmission during the tests mentioned in Figure 13 and Figure 14 are listed in the following Table 3.

The payload length was 22 bytes for GP and 8 bytes for fall and loss of pulse, giving a total of 30 bytes for each packet. These were always transmitted in consecutive packets by the system. The GPS 22 bytes structure can be divided as follows: the first 2 bytes indicate the data channel, the next 2 are the data type, then 4-4-4 are the latitude, longitude and altitude coordinates. These were encoded in the 16-bit number system with an IPSO format.

The signal-to-noise ratio and packet loss rate were different at different measurement locations. On flat terrain, in an undeveloped, sparsely wooded area (Margit island), the signal-to-noise ratio based on 700 transmitted packets was 4.743 dB on average at a distance of 1500 m (where 665 packets were successfully received), and 6.435 dB at a distance of 750 m (where 636 packets were successfully received). It is also important to mention that the packet loss rate at the same location was 9.119% at 1500 m and 4.765% at 750 m. 700 packets were transmitted through the device at both distances. In an urban environment (Óbuda University Tavaszmező street—József boulevard (500 m), Óbuda University Tavaszmező street—Kálvin square (1300 m)) at a distance of 1300 m, the signal to noise ratio was 1.408 dB (receiving a total of 607 packets), and at 500 m, it was 1.919 dB (receiving a total of 642 packets). Similarly, 700 packets were transmitted over both distances, giving a packet loss rate of 13.285% and 8.143% at 1300 [m] and 500 [m], respectively.

The GPS positioning unit in the LoRa module was also tested. Locations with known coordinates in Hungary were selected from the National GPS Network, which contains the coordinates of 1166 locations measured with an accuracy of 2 cm. To validate the measurement, data were requested from the Lechner Knowledge Center [44], which included certain points on Gellért Hill, Normafa and Margaret Island. The position was determined by the LoRa module built into the wristband with a difference of 1.8–2.1 m compared to the known coordinates. The device’s data sheet mentions an accuracy of 2.5 m. The measurements were performed in clear weather, away from built-up areas, which enabled the device to achieve a more accurate result than the value stated in the datasheet. The coordinates obtained by the module were also compared versus a smartphone location system, where the results were generally the same.

**Figure 15:**
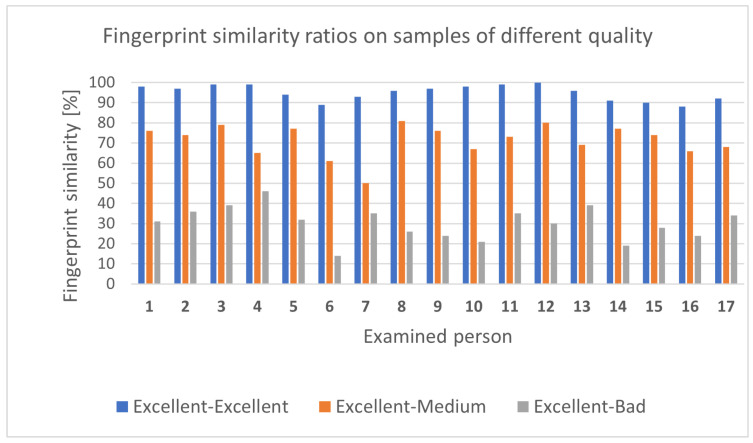
Fingerprint similarity percentages for samples of different qualities [18].

**Fingerprint recognition test:** To test the fingerprint identification system, samples were taken from 17 people, represented in the bar chart in Figure 15. A total of 3 samples from each user were taken, one excellent, one medium, and one poor-quality sample. In the case of the high-quality sample, the sensor and the person’s finger were also cleaned and the person placed the tested finger in the center of the sensor. In the case of the medium-quality sample, the surface of the sensor was not cleaned and the position of the finger was not taken into account. In the case of the poor-quality sample, neither the finger nor the sensor surface was clean, and the examined finger was often only partially fitted in the center of the sensor. From the similarity percentages displayed in Figure 15, it is clear that the system offers reliable fingerprint identification for two high-quality fingerprints. On the other hand, tuning a similarity threshold over a value where the result can be considered a match will be the subject of further research to improve the reliability of the identification process.

**Figure 16:**
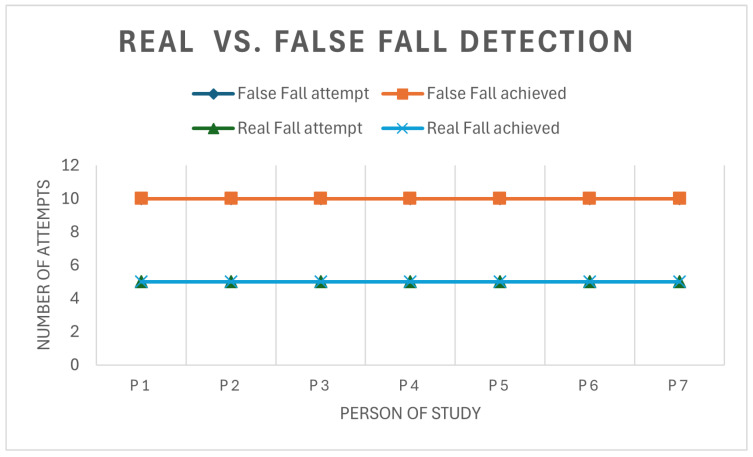
Real vs. False fall discrimination test.

**Fall detection test:** To evaluate the fall detection system, several tests were performed on multiple individuals. A total of 7 people performed at least 10 false falls (sitting down, lying down, climbing stairs, turning around, jumping, walking) and 5 true fall effects (falling into a gymnastic mattress). The test results yielded 100% accuracy, meaning that false falls were not confused with real falls. A simple representation of the test results can be seen in the following graph Figure 16.

**Heart rate module test [45,46]:** The Heart Rate 8 Click is built into the inner bottom of the armband. Thus, the measurement is performed on the lower part of the upper arm. For this reason, these tests were performed on this part of the body. The results were compared to a Xiaomi MiBand 4 bracelet, which was found to be reliable. In the end, a maximum deviation of ±2 BPM was experienced. A total of 30 test measurements were performed and compared.

**Figure 17:**
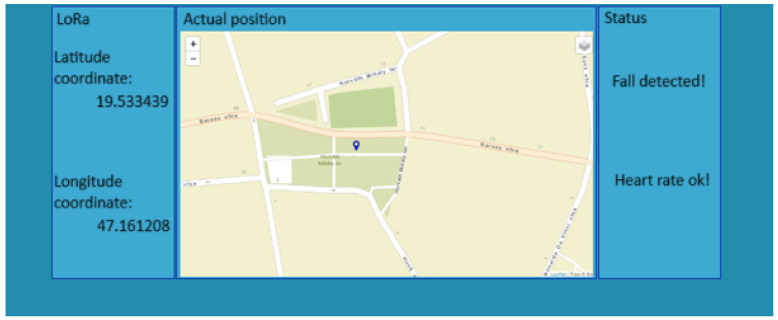
User interface [18].

A Node-RED user interface was implemented for the armband, where the coordinates of the wearer of the device are shown together with the location marked on the map, the status of the heart rate, and the status of the fall, as shown in Figure 17, where the location coordinates are updated once per minute.

**Whole device test:** The finished armband was also tested. This included a 24 h test two times and a 72 h test one time. During this time, the complex operation of the device was evaluated, which included fall detection with an alarm message, heart rate loss with an alarm message, and location control at several times of the day. All functions worked as expected. The device was unable to meet the 72 h operating time; as mentioned in Section 2, the battery is exhausted after 61 h of use. There were 47 usage sessions from full charge to full discharge. The device operated on a battery for an average of 64.41 h. The average is lowered by the fact that in some cases power consumption increases due to testing the vibro motor. The tests elucidated several limitations of the prototype. A limitation of the communication system is that if there is no suitable device to receive the data sent by LoRa, the device is unable to send messages regarding vital signs and coordinates. It is imperative to test the fingerprint sensor on elderly individuals, given the potential for fingerprint deformation, and on individuals of different races, due to the possibility of errors resulting from skin color differences. Additionally, new tests are necessary to account for skin color variations in the calibration of the heart rate sensor, as a higher pigment number could potentially distort the measurement.

## 4. Comparison

It has been mentioned earlier that there are several similar products on the market currently available. An examination of a comparison between the features and capabilities of the armband proposed in this development, can be found in the following Table 4.

In the same way, the position-tracking feature of the proposed device can be compared with currently available wearable devices, offering a wide range of technologies for various purposes, including fitness tracking and location monitoring [51]. While commercially available devices like smartwatches and fitness trackers provide valuable insights [52], their accuracy can vary depending on factors such as GPS signal strength and environmental conditions, as stated in Section 1, e.g., weather conditions and number of satellites connected. Table 5 describes a comparison between currently available devices that match the criteria of wearable location-tracker and the proposed device developed in this paper.

The device discussed in this work is primarily aimed at the elderly, who often do not carry a cell phone with them or cannot handle it at the level that such an armband requires. Therefore, the HealthCare and Safety armband 2020 has an advantage over its competitors, as LoRa communication allows messaging without a mobile phone. Furthermore, none of the devices listed in the previous Table 4 are suitable for biometric identification, while the HealthCare and Safety armband 2020 has an embedded biometric identification system. This allows the armband to be integrated into a home care system and used within a nursing home. This article showed the development process for a wearable device related to elderly care. It should be noted that the main intention was not to directly compare the capabilities of the proposed system with those of the technologies mentioned, i.e., Table 4 and Table 5. In Hungary, the use of technology in elderly care is still in its infancy, making awareness of these systems a crucial priority. On the other hand, additional features such as notification speed or device connectivity consistency are not less important and require to be the subject of further investigation and analysis, which will be considered as part of future improvements. In the case of further development of the device, the design of the PCB is the next step, which makes this armband wearable even on the wrist. Further plans also include a Plastic Logic E-Paper display, as it can follow the wrist’s line due to its flexibility [56].

**Figure 18:**
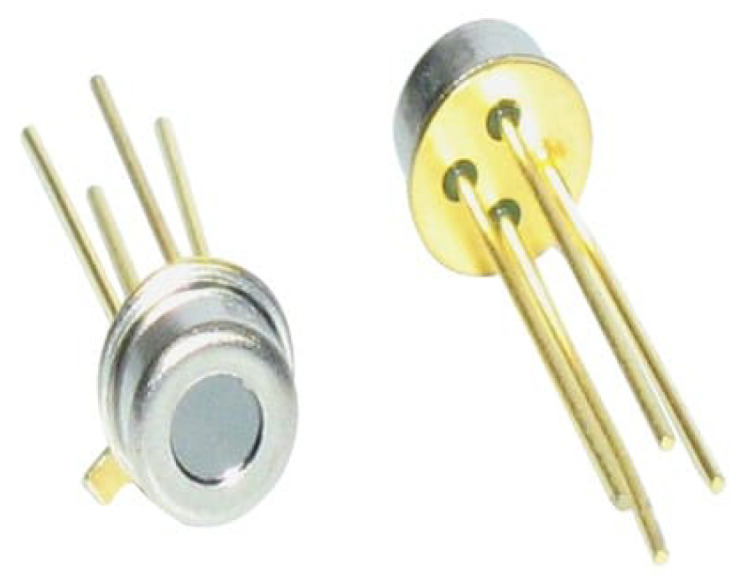
TE Connectivity G-TPCO-031 analog temperature sensor [57].

Using such a display would provide the HealthCare and Safety armband 2020 with similar user interfaces as the other devices mentioned in the table. In addition to these, future work will include the implementation of body temperature sensing, which expands the possibilities of using the armband. To do this, a non-contact device will be used that works based on the infrared principle. The tool would increase the number of biological parameters that can be monitored. Furthermore, none of the devices in the above comparison have such a function, although a change in body temperature can predict a great deal if we can measure it with sufficient accuracy. For this task, it is recommended to use the TE Connectivity G-TPCO-031 analog temperature sensor, which is a device often used in medical devices due to its high accuracy [57]. This sensor is shown in Figure 18.

## 5. Conclusions

The present research proposes the development of an accurate location tracking system for the elderly. Such a device is of particular importance for individuals with dementia and Alzheimer’s disease, who frequently leave their homes and are unable to find their way back. The device is of paramount importance in preventing individuals from becoming lost and in reducing the risk of injury to patients, particularly those suffering from age-related illnesses. The proposed HealthCare and Safety armband 2020 is equipped with an integrated biometric identification system. This allows the armband to be integrated into a home care system and used within a nursing home. Results from the tests described in previous sections were highly satisfactory, demonstrating that the armband functionally fulfills the desired design parameters. During the 24 h and 72 h tests, all functions of the device were tested (i.e., PPG measurement, drop detection, positioning, messaging, fingerprint identification). In general, the results showed a transmission signal-to-loss ratio as low as 4.765% in favorable conditions, fingerprint detection and recognition achieved a 100% hit-rate in high-quality samples, the GPS location tracker showed an accuracy of +/−2 [m], and the battery real working cycle provided an average of 61 active hours. The proposed solution may be of benefit to healthcare professionals, supported housing providers, or simply the elderly and their family members. It is evident that further steps are being taken to enhance the armband, including the optimization of the PCB design, which serves to increase reliability and reduce power consumption.

## Figures and Tables

**Table 1:** LoRa GPS message and decoding.

Payload (Hex)	01 88 06 76 5f f2 96 0a 00 03 e8	
Data channel	Type	Value
01 –>1	88 –>GPS	Latitude: 06 76 5f –>42.3519
		Longitude: f2 96 0a –>−87.9094
		Altitude: 00 03 e8 –>10 m

**Table 2:** Current consumption of devices included in the armband.

Feature	Consumption [mA]
STM32 L412KB	14
Heart Rate 8 click	15.2
GY-521	5.4
LoRa module (with 50 s sleeping)	4.85
Vibro motor 2 click	21.445 (during a notification event)
Operation indicator LED (Flashes for 0.5 s in a frequency of 1/5 Hz)	Aprox. 20
SD Card	38.217 (Electronically isolated when not in use)

**Table 3:** LoRa exploited transmission parameters.

Parameter	Value
**SF**	12
BW	125
ACK	FALSE
FREQ	868,500,000
FCNT	97
CR	4/5
Power Out	14 dBm

**Table 4:** Comparison of armbands currently on the market [47,48,49,50].

Name/Property	Battery Life	ECG	PPG	Fall Detection	Communication Protocol and Distance	GPS	User Interface	Biometrics Identification
HealthCare and Safety armband 2020	Approx 2.5 day	NO	YES	YES	LoRaWAN –>3–15 km from receiver	YES	On PC	YES
Xiaomi MiBand 5	Approx 14 day	NO	YES	NO	Bluetooth –>10–30 m from receiver	NO	Display and phone	NO
Omron HeartGuide BP8000 -M	Approx 2 day	NO	NO	NO	Bluetooth –>10–30 m from receiver	NO	Display and phone	NO
Philips Health Watch	Approx 4 day	NO	YES	NO	Bluetooth –>10–30 m from receiver	NO	Display and phone	NO
Garmin Vívoactive 4S	Approx 7 day	NO	YES	NO	Bluetooth, ANT+, WiFi –>max 100 m from receiver	YES	Display and phone	NO
Asus VivoWatch SP HC-A05	Approx 10 day	YES	YES	NO	Bluetooth –>10–30 m from receiver	YES	Display and phone	NO

**Table 5:** Location tracking comparison with available devices.

Device Name	Min. Accuracy [m]	Max. Accuracy [m]
HealthCare and Safety armband 2020	1.8	2.1
Smartwatches [53] (Apple Watch, Samsung Galaxy Watch, Fitbit Versa)	3	10
Fitness Trackers [52] (Fitbit Charge, Garmin Vivosmart)	3	10
GPS Trackers [54] (AngelSense, Tile Mate)	2	5
Personal Locator Beacons [55] (PLBs)	3	10

## Data Availability

Data is available upon request.
